# Factors Affecting Perceived Stigma in Leprosy Affected Persons in Western Nepal

**DOI:** 10.1371/journal.pntd.0002940

**Published:** 2014-06-05

**Authors:** Bipin Adhikari, Nils Kaehler, Robert S. Chapman, Shristi Raut, Paul Roche

**Affiliations:** 1 TB/HIV Department, Medecins Sans Frontieres Holland, Nasir Hospital, Nasir, South Sudan; 2 College of Public Health Sciences, Chulalongkorn University, Bangkok, Thailand; 3 Rissa Legesenter, Rissa, Norway; 4 Department of Microbiology, Manipal College of Medical Sciences, Pokhara, Nepal; 5 Green Pastures Hospital and Rehabilitation Centre, Pokhara, Nepal; Common Heritage Foundation, Nigeria

## Abstract

**Background:**

There are various factors which construct the perception of stigma in both leprosy affected persons and unaffected persons. The main purpose of this study was to determine the level of perceived stigma and the risk factors contributing to it among leprosy affected person attending the Green Pastures Hospital, Pokhara municipality of western Nepal.

**Methods:**

A cross-sectional study was conducted among 135 people affected by leprosy at Green Pastures Hospital and Rehabilitation Centre. Persons above the age of 18 were interviewed using a set of questionnaire form and Explanatory Model Interview Catalogue (EMIC). In addition, two sets of focused group discussions each containing 10 participants from the ward were conducted with the objectives of answering the frequently affected EMIC items.

**Results:**

Among 135 leprosy affected persons, the median score of perceived stigma was 10 while it ranged from 0–34. Higher perceived stigma score was found in illiterate persons (p = 0.008), participants whose incomes were self-described as inadequate (p = 0.014) and who had changed their occupation due to leprosy (p = 0.018). Patients who lacked information on leprosy (p = 0.025), knowledge about the causes (p = 0.02) and transmission of leprosy (p = 0.046) and those who had perception that leprosy is a severe disease (p<0.001) and is difficult to treat (p<0.001) had higher perceived stigma score. Participants with disfigurement or deformities (p = 0.014), ulcers (p = 0.022) and odorous ulcers (p = 0.043) had higher perceived stigma score.

**Conclusion:**

The factors associated with higher stigma were illiteracy, perceived economical inadequacy, change of occupation due to leprosy, lack of knowledge about leprosy, perception of leprosy as a severe disease and difficult to treat. Similarly, visible deformities and ulcers were associated with higher stigma. There is an urgent need of stigma reduction strategies focused on health education and health awareness programs in addition to the necessary rehabilitation support.

## Author Summary

A total of 135 leprosy affected persons were interviewed with a questionnaire containing EMIC questions designed to assess the level of perceived stigma and the questionnaire containing variables for socio-demographic characteristics, knowledge about leprosy and the clinical presentations of the participants. Clinical presentation as disability was graded according to WHO guidelines, where grade 0 means no disability found, grade I means loss of sensation has been noted in the hand or foot while grade II means visible damage or disability. Total EMIC score was analyzed between sub-variables to see the factors associated with the higher level of perceived stigma score. Additionally, among the total participants, we included 20 of them who were admitted at hospital for various reasons. Two sets of focus group discussions were conducted with additional questions to derive the reasons behind frequently affected EMIC stigma domains. The factors associated with higher perceived stigma score were illiteracy (those who could not read and write), perceived economical inadequacy, lack of knowledge on leprosy, the perceptions as difficult to treat and severe disease and presence of visible deformities and ulcers. Considering our findings pertaining to higher perceived stigma, there is an urgent need of stigma reduction strategies which should focus on health education about leprosy that can change the perceived stigma in leprosy.

## Introduction

Leprosy is a chronic granulomatous disease caused by *Mycobacterium leprae*. Besides clinical sequel followed usually after infection, the consequences of stigma associated with leprosy outweigh the burden of physical afflictions [1]. Three kinds of stigma associated with leprosy affected persons have been described. Experienced or enacted stigma refers to the real discrimination or acts experienced by leprosy affected persons while perceived stigma refers to the development of fear within an affected person where the fear may arise out of potential discrimination from family members, friends or society. As a consequence of both enacted and perceived stigma, a person over a long period of time may believe what others think and say about him, resulting to the loss of self-esteem and dignity which is referred to be a self-stigma or internalized stigma [2].

Stigma affects the psychosocial well-being of the affected person. A person may feel fear or shame which can lead to anxiety and depression. The resultant anxiety and depression may lead to decreased social participation and social exclusion [3].

Anticipation of stigma may cause affected person to conceal their condition [4]. The burden of keeping this secret, of being ever watchful and careful takes an emotional toll and adversely affects health seeking behavior [3]. Concealing the disease, avoiding the questions regarding the disease and at times even telling lie for the fear of disclosure was found to be a major concern for leprosy affected persons attending Green Pastures Hospital, Nepal [5].

Stigma has been found to be associated with misconceptions about the disease, visible deformities and the development of ulcers [4]. Disability is a broad term covering any impairment, activity limitation or participation restriction affecting a person. According to WHO, grade 0 means no disability is found. Grade I means that loss of sensation has been noted in the hand or foot while grade II means the visible damage or disability is noted [6]. Visible deformities and disabilities have been found to be the prominent contributor of stigma development in leprosy affected persons [7] while it also triggers the development of negative attitudes towards leprosy among unaffected people [8].

In a systematic review of risk factors contributing to stigma, the basis of stigma development was found to be the visibility of the disfigurements and disability augmented by the stereotypes of the society, knowledge and the status of the person in terms of economy, education and ability to participate in society [9].

In Nepal, leprosy is still a stigmatizing disease. Misconceptions about the disease have contributed to the development of negative attitudes to leprosy affected persons. In a study conducted in eastern Nepal, fear of infection and god's curse were found to be the most prevalent causes of negative behavior towards leprosy affected persons [8]. In the other study [10] conducted in eastern part of Nepal, the causes of stigma perception in leprosy affected persons were consistent with the causes of negative attitudes in unaffected community members [8]. The beliefs and perceptions about leprosy were found to be the prominent causes of stigma [10]. Fear of infection, was the most important cause of stigma different countries including China [11] and India [12]. In India, in addition to the fear of infection, false beliefs about leprosy, ignorance about the disease and lower socio-economic status were associated with stigma in leprosy [12]. Therefore, we hypothesized that there is association between the levels of perceived stigma in leprosy affected persons and the factors characterizing them (demographic characteristics, knowledge about leprosy, natural history of disease, clinical presentation, disability grades and reaction) While few studies are done in eastern part of Nepal, most of them are focused on the impact of the stigma, participation restriction and income generation. There has been no research so far in leprosy stigma in a view to explore the factors associated with it. The specific objective of this study was to determine the prevalence of perceived stigma and its association with factors such as socio-demographic, knowledge about leprosy and clinical presentation characterizing leprosy affected persons attending Green Pastures Hospital and Rehabilitation Centre.

Green Pastures Hospital and Rehabilitation Centre, the only known leprosy referral center in western region of Nepal provides the services for leprosy patients with disability management, treatment and vocational training. Therefore, exploring the risk factors of stigma in leprosy affected persons attending GPH&RC can help to understand the leprosy stigma and therefore can direct the stigma reduction strategies and intervention programs.

## Materials and Methods

The study was cross-sectional in design. The study population comprised leprosy affected people attending Green Pastures Hospital and Rehabilitation Centre, specific for the treatment of leprosy and rehabilitation in western region of Nepal. The study sample comprised people affected by leprosy who were undergoing treatment for leprosy reactions, ulcers, disability-rehabilitation in addition to self-care training at Green Pastures Hospital and Rehabilitation Centre. All participants were included in the study who visited the hospital between the periods of February 2013 to March 2013. Considering the limited number of people affected by leprosy visiting the hospital, pilot testing of the questionnaire was not conducted.

Total 135 leprosy affected persons were interviewed using a questionnaire containing socio-demographic characteristics (age, sex, ethnicity, marital status, location, type of family and leprosy affected persons in family/relatives/neighbors), socio-economic conditions (occupation, income, nature of work, job, education and religion), Knowledge about leprosy (information about leprosy, cause of leprosy, infectiousness, transmission, treatment, signs and symptoms about leprosy) and Clinical presentation of leprosy (ulcer, disfigurement, deformity and disability status). Clinical conditions such as ulcer, disfigurement, deformity and disability grades were obtained from the hospital treatment card which individual participants carried with them. Knowledge about leprosy was assessed using questions with answers as yes or no and corresponding sub-questions on further knowledge regarding the particular items.

In addition, the EMIC scale questionnaire was asked to each participant. The EMIC scale has been developed to elicit illness-related perceptions, beliefs and the practices [13]. The EMIC questionnaire has 15 items related to perception of stigma in leprosy and has been validated and shown to be reliable in a study in India [14]. EMIC scale has been available in different languages including Nepali language and is the recommended instrument in terms of measuring leprosy related stigma. Higher the score obtained by EMIC scale higher is the level of perceived stigma. It has been classified as the instrument to measure the perceived stigma in leprosy by The International Federation of Anti-Leprosy Association (ILEP) and the stigma research workshop held in Amsterdam in 2010 [15], [16].

Descriptive statistics were used to describe the socio-economic, knowledge level and clinical presentation of the participants. Difference in total perceived stigma score using EMIC between different categorical variables were analyzed using Mann Whitney U test and Kruskal Wallis H test since these scores were not normally distributed.

Focus group discussions were conducted with 20 people affected with leprosy who were admitted in ward. Semi-structured questionnaire were designed to explore the deeper reasons for the most affected EMIC domains which were reasons of concealment, lower self-esteem, less respect from others, impacts on marriage and their experiences with leprosy. Out of 42 people who were admitted in ward during that period, only 20 of them agreed to participate in 2 sets of focus group discussion each containing 10 participants. An oral consent was taken with each participant before they took part in discussion.

Both sets of focus group discussion were recorded in mobile phone recorder later typed into computer. Different themes according to the questionnaire were segregated and frequencies of themes were based as evidence for the formulation of conclusion.

### Ethics statement

Ethical permission for this research was obtained from Nepal Health Research Council and International Nepal Fellowship Research Committee. People were eligible if they were affected by leprosy, age above 18 years and willing to participate. Interviews were only conducted after the written consent was received and was conducted by principal investigator. Interviews were conducted with all leprosy affected people attending GPH&RC from February 2013 to March 2013. Attempt was done to include equal number of participants from the ward and OPD, 5 from the ward and 3 from the OPD denied the written consent, however, there were no drop outs. The interviewer taking into the consideration the sensitivity of the subject established a friendly rapport before the interview and encouraged participants to express their views. The anonymity of the participants was secured by coding the participants' name. No incentives were offered or paid for their time.

## Results

### EMIC profile

All participants who met the eligibility criteria were recruited into the study after taking written consent. Total 135 participants were asked with the questionnaire form. Among the questions representing different aspects of perceived stigma in EMIC questionnaire, most affected areas of perceived stigma were concealment of the disease, self-esteem, disclosure concern and the shame and embarrassment due to leprosy (Table 1). Among the total participants 65.9% affirmed that they would conceal the disease condition as long as it is possible while 57.8% anticipated decreased self-esteem due to the disease condition and 40.7% only disclosed the disease condition to the close ones.

**Table 1:** EMIC profile of leprosy affected persons answering yes (n = 135).

Items	Number (%)
Keep others from knowing if possible	89(65.9%)
Think less of yourself because of this problem	78(57.8%)
Disclosed to the close person about this condition	55(40.7%)
Shame or embarrassment due to Leprosy	50(37%)
Less respect from others because of this problem	37(27.4%)
Others have avoided you because of this problem	27(20%)
Causes marriage problems to your family	26(19.3%)
Others would think less of your family	25(18.5%)
Others might refuse to visit your home	17(12.6%)
Decided on self to stay away from social group	17(12.6%)
Others presume you have other health problems	17(12.6%)
Contact with you would have bad effects on others	16(11.9%)
Social problems to your children in community	13(9.6%)
Disease causes problems for your marriage	12(8.9%)
Asked to stay away from work or social group	4(3%)

### Socio-demographic characteristics

Of the 135 leprosy affected participants, 58.5% of them were those who attended OPD at the hospital. Total median score of EMIC scale was higher among those leprosy patients who were in the ward compared to those who attended OPD (p = 0.006). There was no significant difference in mean EMIC score between different age groups (p = 0.199), sex (p = 0.344), ethnicity (p = 0.934), location (p = 0.072), marital status (p = 0.477) and family type (p = 0.356). Similarly, participants were asked if they had any other member of their family affected by leprosy in past or present including if they had relatives or neighbors affected by leprosy. Neither of them had significant difference in median score of stigma (Table 2). There was a significant difference in median EMIC score (p = 0.008) between different level of education in participants classified as illiterate (those who could not read and write), those who attended primary level (<5 years of education) and those who attended secondary and higher education (>5 years). On post hoc analysis, the illiterate and those who attended more than 5years of education had significant difference in median score (p = 0.03). Similarly, when EMIC scores among subjects with less than 5 years education were compared with those with more than 5 years there was a significant difference (p = 0.016) while EMIC scores of the illiterate and those who attended <5 years of education were not significantly different (p = 0.673). There was no significant difference in median score between religious groups Hindu and other (p = 0.309), Occupation (p = 0.321), and amount of income (p = 0.068). However, on post hoc analysis two different income groups (the highest and lowest income group) showed significant difference (p = 0.011) There was a significant difference in EMIC score between those who felt economic inadequacy and who did not (p = 0.014). Similarly, there was also significant difference in stigma score between those who had to change their occupation after being affected by leprosy and those who did not (p = 0.018).

**Table 2:** Socio-demographic characteristics in relation to the EMIC score (n = 135).

Characteristics	Number (%)	Median	P-value
**Source of patients (n = 135)**			
OPD	79(58.5)	9	**0.006**
Ward	56(41.5)	14.5	
**Age Groups (n = 135)**			
34 years or below	28(20.7)	9	0.199
35–54 years	39(28.9)	12	
55–64 years	40(29.6)	11.5	
65 years or above	28(20.7)	9.5	
**Sex (n = 135)**			
Female	50(37)	10	0.344
Male	85(63)	10	
**Ethnicity (n = 135)**			
Brahmin	26(19.3)	9.5	0.934
Chhetri	29(21.5)	9	
Gurung	11(8.1)	9	
Magar	20(14.8)	12.5	
Other	49(36.3)	10	
**Location (n = 135)**			
Western region	117(86.7)	10	0.072
Mid-west/far west and central	18(13.3)	17.5	
**Marital status (n = 135)**			
In relationship	104(77)	11	0.477
Not in relationship	31(23)	9	
**Family Type (n = 135)**			
Joint family	108(80)	10	0.356
Nuclear family	27(20)	11	
**Leprosy affected in family (n = 135)**			
Yes	34(25.2)	9	0.19
No	101(74.8)	11	
**Leprosy affected in relatives/neighbors (n = 135)**			
Yes	23(17)	12	0.549
No	112(83)	10	
**Level of Education (n = 135)**			
Illiterate	74(54.8)	11	**0.008**
Primary education (<5 years)	33(24.4)	12	
Secondary and higher (>5 years)	28(20.7)	7	
**Religion (n = 135)**			
Hindu	107(79.3)	11	0.309
Other	28(20.7)	7.5	
**Occupation (n = 135)**			
Farmer	76(56.3)	11	0.321
Unemployed	16(11.9)	13	
Other	43(31.9)	9	
**Amount of Income (n = 135)**			
≤4000 NRS	40(29.6)	14	0.068
4001–8000 NRS	53(39.3)	10	
8001–12000 NRS	23(17)	9	
≥12001 NRS	19(14.1)	8	
**Enough to sustain living (n = 135)**			
Yes	90(66.7)	9	**0.014**
No	45(33.3)	14	
**Change of Occupation (n = 135)**			
Yes	63(46.7)	13	**0.018**
No	72(53.3)	9	

### Knowledge about leprosy and perceived stigma score

Knowledge and perceptions about leprosy and perceived stigma scores were analyzed in all participants. The overall stigma score for those who had knowledge about leprosy was lower than those who lacked knowledge of leprosy (Table 3). There was a significant difference in EMIC stigma score between those who had information on leprosy (p = 0.025), knowledge on leprosy cause (p = 0.02) and knowledge on transmission (p = 0.046). Similarly, participants who did not have knowledge of leprosy signs and symptoms had lower stigma scores compared to those who knew one or more signs and symptoms of leprosy although this was statistically insignificant (p = 0.344). There was a difference in EMIC stigma score who perceived leprosy as a very infectious disease (p = 0.127). Similarly, there was a significant difference in perceived stigma score between groups who felt that leprosy is difficult to treat (p<0.001) and a severe disease (p<0.001).

**Table 3:** Knowledge about leprosy in relation to EMIC score (n = 135).

Characteristics	Number (%)	Median	P-value
**Information on Leprosy (n = 135)**			
Yes	39(28.9)	8	**0.025**
No	96(71.1)	12	
**Knowledge on Leprosy cause (n = 135)**			
Yes	52(38.5)	8	**0.02**
No	83(61.5)	12	
**Knowledge on transmission (n = 135)**			
Yes	51(37.8)	9	**0.046**
No	84(62.2)	12	
**Knowledge on sign and Symptoms (n = 135)**			
Don't know	48(35.6)	12	0.344
Single	28(20.7)	9	
Multiple	59(43.7)	9	
**Leprosy is very infectious (n = 135)**			
Yes	27(20)	13	0.127
No	108(80)	9.5	
**Difficult to treat (n = 135)**			
Yes	48(35.6)	14	**<0.001**
No	87(64.4)	9	
**Severe Disease (n = 135)**			
Yes	81(60)	12	**<0.001**
No	54(40)	8	

### History of disease, clinical presentation and perceived stigma score

Brief history of disease and clinical presentations were asked and assessed respectively with all the participants (Table 4). Participants' age at diagnosis (p = 0.213) and years after diagnosis (p = 0.967) did not show any difference in EMIC score. First sign and symptoms were categorized into skin involvement, nerve involvement and deformity development. Neither of them showed significant difference in perceived stigma score (p = 0.792). Similarly, there was no significant difference in EMIC between participants who sought hospital or doctor soon after development of signs and symptoms and who did not (p = 0.079). The majority (55.6%) of participants received first treatment from non-medical providers such as witch doctors and traditional healers. There was no significant difference in EMIC score between groups of participants who received treatment from medical providers, non-medical providers and friends/family and others (p = 0.255). Similarly, there was no significant difference in EMIC score between those who completed treatment and who did not (p = 0.156). There was a significant difference in EMIC score in participants who had disfigurement or deformities (p = 0.014), ulcer (0.022) and odorous ulcer (0.043) compared to those who did not. However, there was no significant difference in EMIC between those who had reaction and who did not (p = 0.331).

**Table 4:** History of disease and clinical presentation in relation to EMIC score (n = 135).

Characteristics	Number (%)	Median	P-value
**Age at Diagnosis**			
≤20 years or below	42(31.1)	11.5	0.213
21–40 years	41(30.4)	8	
41–60 years	37(27.4)	12	
61 years or above	15(11.1)	9	
Mean = 35.05, Median = 33.00			
SD = 18.52, Range = 7–77			
**Years after Diagnosis**			
≤20 years or below	95(70.4)	10	0.967
21–40 years	19(14.1)	10	
41 years or above	21(15.6)	13	
Mean = 15.20, Median = 5.00			
SD = 18.57, Range = 0–66			
**First sign/symptom (n = 135)**			
Skin Involvement	82(60.7)	10	0.792
Other(Nerves and Deformity)	53(39.3)	10	
**Sought hospital or doctor (n = 135)**			
Yes	69(51.1)	9	0.079
No	66(48.9)	12.5	
**First treatment from (n = 135)**			
Medical Providers	33(24.4)	11	0.255
Non-medical Providers	75(55.6)	9	
Friends/family and others	27(20)	13	
**Stage of Treatment (n = 135)**			
RFT (Released From Treatment)	88(65.2)	12	0.156
Undergoing Treatment	47(34.8)	9	
**Do you have disfigurement or deformities (n = 135)**			
Yes	67(49.6)	13	**0.014**
No	68(50.4)	9	
**Did you ever have Ulcer (n = 135)**			
Yes	75(55.6)	13	**0.022**
No	60(44.4)	9	
**Odorous ulcer (n = 75)**			
Yes	30(40)	17	**0.043**
No	45(60)	9	
**Did you ever have Reaction (n = 135)**			
Yes	79(58.5)	9	0.331
No	56(41.5)	11	

### WHO disability grading and perceived stigma scores

More than half (51.1%) of the participants had grade II disabilities and higher EMIC stigma score compared to grade 0 and grade I disabilities (p = 0.161) (Table 5). However, the difference in EMIC stigma score showed marginal significance between grade II and grade 0 combined with grade I (p = 0.056), not shown in table.

**Table 5:** WHO disability grading in relation to EMIC score (n = 135).

Characteristics	Number (%)	Median	P-value
Grade 0	28(20.7)	9	0.161
Grade I	38(28.1)	9	
Grade II	69(51.1)	12	

### Focus group discussion

In majorities of the leprosy affected persons as evident from EMIC profile, concealment of the disease, lowered self-esteem and the disclosure to the close ones were major aspects of the EMIC questionnaire which contributed to higher EMIC score compared to the marital problems, social exclusion acts and impacts to their family members. Focus group discussion with leprosy affected persons concluded that the discrimination and stigma attached to the disease was felt to be decreasing over the time. However, the reasons for most of the participants' intention not to disclose their disease condition were the fear of discrimination, isolation and rejection. The most often reported cause of fear was the strongly rooted stereotype attached to the disease. The most common belief leprosy affected person presumed was the fear of transmission of the disease among others.

In addition to the prevalent false beliefs about the transmission, severity and myths attached with the disease, the deformities and ulcers were also reported to be the triggering factor for the disease disclosure. While most of the participants realized that ulcers and disabilities due to leprosy were affecting them physically, its psychosocial burden was the greater problem. Some patients never reported to their close ones about their causes of disabilities and ulcers. Instead they often told the causes of disabilities and ulcers to be due to some other disease. However, participants realized that keeping this secrecy was a huge burden for them.

## Discussion

In this study, the EMIC scale was used to measure the level of perceived stigma and its association with socio-demographic conditions, clinical conditions and level of knowledge about leprosy. Different aspects of perceived stigma were assessed using EMIC scale. Concealment, disclosure and self-esteem were the major domains found to be more frequently affected than other. 66% of Leprosy affected persons intended to conceal their disease and 59% never told their close relatives. 58% experienced lowered self-esteem due to leprosy. These findings suggest that perceived fear after disclosure was significant than the other aspects of EMIC which were more related to real life situations. This is consistent with studies conducted in Eastern Nepal [4], study in Northern India [17] and study in Netherland [18].

In this study, the level of perceived stigma score was higher in in-patients compared to those who attended OPD. As most of the ward patients were admitted for the treatment of ulcer, reactions and rehabilitation, this might have been one of the reasons for them to experience more perceived stigma compared to OPD patients. The higher perceived stigma score was found in illiterate and those who had less than 5 years of education compared to those who had education for more than 5 years. This is consistent with a study done in India [19]. The impact of education on perceived stigma score could be to increase the overall knowledge on disease and an increased ability to resist the negative stereotypes attached to the disease. Similarly, economic inadequacy and the lower income group participants showed significantly higher perceived stigma compared to those who had higher income. The association of poor economic conditions and higher level of stigma has been consistent with the studies in Bangladesh [20] and India [19]. Change in occupation in leprosy affected persons has been one of the grave consequences of disability caused by leprosy. However, change in occupation could be due to leprosy diagnosis alone. This study showed higher perceived stigma in those who were obliged to leave their occupation. This was consistent with a study done in Eastern Nepal [21].

Knowledge about leprosy was assessed regarding the information on leprosy, leprosy cause, transmission, signs and symptoms and the perception of the disease in terms of infectiousness, treatment and severity. Those who lacked information on leprosy, knowledge about leprosy cause, leprosy transmission and those who had perceptions (difficult to treat, and a severe disease) had higher EMIC score compared to those who did not. These findings have been consistent with a study done in Eastern Nepal [10] and India [12]. While lack of knowledge regarding leprosy and the false perceptions were found with more perceived stigma in leprosy affected persons, this was equally true with the unaffected population in Western Nepal where these factors were associated with higher level of perceived stigma [22]. In a study conducted in Eastern Nepal, the false perceptions about the disease transmission, contagiousness was found to be the major cause of community stigma [8]. The negative perceptions and association of stigma was evidenced in different other studies including China [11] and Africa [23].

Leprosy affected persons with visible impairments or deformities had higher EMIC score compared to those who did not. In this study, WHO grade II disabled patients had higher stigma compared to grade 0 and grade I, however, it was statistically insignificant. This has been consistent with a study done in Eastern Nepal [7]. Similarly, our finding on disabilities has been consistent with a study done in Brazil where level of stigma was higher in grade II disabled patients compared to grade 0 and grade I [24]. Disabilities have been found to be associated with leprosy stigma in number of studies in different parts of the world [12], [19], [20], [23], [25], [26]. Ulcer affected persons in our study had higher stigma score compared to those who did not followed by the sub sample of ulcer affected persons who had foul odor had higher stigma score compared to those who did not. The reason for higher stigma in ulcer affected persons could be because of the visibility of the wound which consequently could trigger the stigma process in both leprosy affected persons and unaffected persons. The ulcer and its association with stigma have been consistent with a study conducted in Nepal [4].

### Limitations

This study was conducted in western region of Nepal, where only those people who visited hospital for treatment, rehabilitation and wound care were recruited while many other people affected by leprosy who did not have any symptoms were not included in the study which limits our finding to generalize over all leprosy affected persons. Only perceived stigma was assessed in this study while two other types of stigma were not assessed therefore, stigma in this study cannot be the whole picture of stigma. While clinical presentations of the participants were obtained from the hospital treatment card, many other questions might have encountered recall biases. The full evaluation of the data using multiple regressions was not done in this study which could have strengthened our findings.

### Conclusion

This study concludes that lower education level, perceived economic inadequacy, obligation to change the occupation due to leprosy, lack of knowledge and the wrong perceptions about leprosy were the significant factors contributing to higher levels of perceived stigma in leprosy affected persons. In addition to these socio-demographic factors, the presence of visible deformities, ulcers and disabilities also contributed to higher perceived stigma in leprosy affected persons. The major aspects of EMC stigma scale affected were the attitude to conceal the disease, and lowered self-esteem. The major causes for these have been explained by focus group discussion as the perceived fear of discrimination, rejection and the society's fear of transmission.

The factors contributing to the development of stigma in leprosy affected persons from this study can direct the need of intervention programs focusing on health education. Health education which might correct the wrong perceptions and might increase understanding of leprosy and the people affected can have a significant impact in both leprosy affected persons and leprosy unaffected persons. In addition to the education and health awareness programs, empowerment of the leprosy affected persons by technical education, vocational training and social participation might be helpful to increase self-esteem and reduce perceived stigma.

Ulcers and visible deformities have been found as contributing factors for the higher level of perceived stigma. Early case detection through training of health professionals and health education to the general public might prevent the delays in presentation, ulcers, and deformities which ultimately can reduce the stigma.

## Supporting Information

Appendix S1 **Questionnaire for leprosy affected persons.**
(DOCX)

Appendix S2 **Questionnaire for focus group discussion.**
(DOCX)

Checklist S1 **STROBE checklist for cross-sectional studies.**
(DOC)
