# Supplementary material for: Factors Affecting Perceived Stigma in Leprosy Affected Persons in Western Nepal
Source: PLoS Negl Trop Dis. 2014 Jun 5;8(6):e2940. doi: 10.1371/journal.pntd.0002940 (PMC4046961; doi:10.1371/journal.pntd.0002940)
Supplement: Appendix S1 — Questionnaire for leprosy affected persons. (DOCX) [file pntd.0002940.s001.docx]

**Appendix S1: Questionnaire for leprosy affected persons**

A. Socio-demographic characteristics

B. Knowledge about leprosy, natural history of disease, clinical presentation and disability

C. Disability according to the WHO classification and reaction associated with leprosy

D. Explanatory Model Interview Catalogue (EMIC for leprosy affected persons)

**A. Socio-demographic entities:**

1. Name:
2. Age :
3. Sex:
4. Ethnicity:
5. Location:
6. Marital status:
   1. □ Unmarried
   2. □ Married
   3. □ Divorced
   4. □ Separated
   5. □ Widowed
7. Type of family:
   1. □ Joint
   2. □ Nuclear
8. Family affection:
   1. Anybody in family affected by leprosy? □ Yes □ No
   2. Anybody in close relationship affected by leprosy? □ Yes □ No
   3. Anybody in neighbor affected by leprosy? □ Yes □ No
9. Education: Can you read and write? □ Yes (Literate) □ No (Illiterate)

*If yes*, how many years did you study?

1. Primary level (< 5 years)
2. Secondary level (5-10 years)
3. Higher education (> 10 years)
4. Religion: What is your religion?
   1. □ Hindu
   2. □ Buddhist
   3. □ Muslim
   4. □ Christian
   5. □ Other

1. Occupation: What is your occupation?
   1. □ farmer
   2. □ Laborer
   3. □ private business
   4. □ civil/office
   5. □ student
   6. □ house-wife/man
   7. □ unemployed
   8. □ other
2. Who generates income to support Family?
   1. □ Self
   2. □ Father
   3. □ Mother
   4. □ Brother
   5. □ Sister
   6. □ other
3. What is the source of income?
   1. □ Agriculture
   2. □ Governmental Job
   3. □ Private Job
   4. □ Foreign Employment
   5. □ Business
   6. □ Other
4. What is the nature of work?
   1. □ Labor work
   2. □ Non-labor work
5. How much does monthly income amount? (1 US $ is approx. 85 NRS)
   1. □ ≤ 4000 NRS
   2. □ Between 4000 NRS to 8000 NRS
   3. □ Between 8000 NRS to 12000 NRS
   4. □ Between 12000 NRS to 16000 NRS
   5. □ Above 16000 NRS
6. Is that income generation enough to sustain living conditions?
   1. □ Yes
   2. □ No
7. Did you have to change your job because of leprosy?
   1. □ Yes
   2. □ No

**B. Knowledge about leprosy, natural history of disease, clinical presentation and disability:**

1. Did you ever receive information on leprosy?

- - 1. □ Yes
    2. □ No

*If yes,* where did you get the information from (several selections possible)?

1. □ Hospital
2. □ Health centers
3. □ Local health workers
4. □TV/Newspaper/Radio
5. □ Friend or family
6. □ Other

2. Do you know what causes leprosy? □ Yes □ No

*If Yes,* Please choose one of these:

- 1. □ Bacteria or any micro-organism
  2. □ Curse by god
  3. □ Karma
  4. □ Other: specify (_____________)

3. Do you think leprosy is very infectious?

- 1. □ Yes
  2. □ No

4. Do you know how leprosy is transmitted? □ Yes □ No

1. □ From infected person
2. □ From water/soil contamination
3. □ From animal
4. □ From mosquito
5. □ Other: specify (_____________)

5. Do you think leprosy is difficult to treat?

1. □ Yes
2. □ No

6. Do you think leprosy is a severe disease?

1. □ Yes
2. □ No

7. Do you know the signs and symptoms of leprosy? *If Yes,*

1. □ Patches
2. □ Tingling
3. □ Anesthesia
4. □ Deformity
5. □ Ulcer
6. □ Don’t know

8. How old were you when you were diagnosed with Leprosy? ______ (years)

9. What was your first sign/symptom of leprosy?

- 1. □ Skin involvement
  2. □ Nerve involvement
  3. □ Skin and nerve involvement
  4. □ Deformity

10. Did you go to a doctor/hospital as soon as you noticed the first sign/symptom?

- 1. □ yes
  2. □ no

*If no*, why did you not seek for medical help (several selections possible)?

a. □ Had to ask head of the family

b. □ felt ashamed

c. □ Didn’t had problem with the sign(s)/ symptom(s)

d. □ Didn’t know it was Leprosy

e. □ Awaited for self-cure

11. Where did you get your first treatment?

- - 1. □ Primary Health center
    2. □ local health worker
    3. □ Local hospital
    4. □ Tertiary hospital
    5. □ Non-medical professional/healer/witch doctor etc.
    6. □ Friend
    7. □ Other

12. At what stage is patient at present in relation to the treatment?

1. □ RFT (Release From Treatment)
2. □ Undergoing treatment (2months or more)
3. □ Just started treatment ( less than 2 months)

13. If Released From Treatment: How long did you have medicine for?

1. □ 6 months
2. □ 12 months
3. □ 24 months
4. □ Not adhered to complete treatment.

14. Does the patient have disfigurement or deformity?

1. □ Yes
2. □ No

15. When did you develop first disfigurements or deformities?

1. □ Before the first visit to a health center
2. □ During the treatment at a health center
3. □ After the treatment at a health center

16. Was the first disfigurement or deformity visible to others?

1. □ Yes
2. □ No

17. Where was the first disfigurement or deformity?

1. □ Facial region
2. □ Hand and/or foot
3. □ Eyes
4. □ Other body parts

18. Did you ever have ulcer? □ Yes □ No

*If yes,* which part of body did you have ulcer?

1. □ Facial region including eyes and ears
2. □ Hands
3. □ foots
4. □ Other body parts: specify (_____________)

19. Was/Is your ulcer visible to other?

1. □ Yes
2. □ No

20. Was/Is your ulcer smelling foul?

1. □ Yes
2. □ No

21. Can you walk normal? (Please see if patient has abnormal gait)

1. □ Yes
2. □ No

**C. Disability according to the WHO classification:**

| “Grade 0”  □ | **- No disability found**. |
| --- | --- |
| “Grade 1”  □ | - **Loss of sensation** noted in the hand or foot, this does not include the loss of sensation in the skin patch (Eyes are not given grade 1). |
| “Grade 2”  □ | - **Visible damage or disability.** For the eyes, this includes the inability to close the eye fully or obvious redness of the eye, visual impairment or blindness.  - For the hands and feet, visible damage includes wounds and ulcers as well as deformity due to muscle weakness, such as foot drop or claw hand.  - Loss or partial reabsorption of fingers or toes. |

22. Did you ever have Leprosy Reaction? (Cross check with patient’s treatment card)

1. □ Yes
2. □ No

23. When did you have reaction?

1. □ Before the treatment
2. □ During the treatment
3. □ After the treatment (RFT = Release From Treatment)

24. Was/Is Reaction Visible to the other people?

1. □ Yes
2. □ No

**D. Explanatory Model Interview Catalogue (EMIC) stigma scale, adapted for leprosy affected people**

| **No.** |  | **Yes** | **Possibly** | **Uncertain** | **No** | **Score** |
| --- | --- | --- | --- | --- | --- | --- |
|  |  | **3** | **2** | **1** | **0** |  |
| 1. | If possible, would you prefer to keep people from knowing about leprosy? |  |  |  |  |  |
| 2. | Have you discussed this problem with the person you consider closest to you, the one whom you usually feel you can talk to most easily? |  |  |  |  |  |
| 3. | Do you think less of yourself because of this problem? Has it reduced your pride or self-respect? |  |  |  |  |  |
| 4. | Have you ever been made to feel ashamed or embarrassed because of this problem? |  |  |  |  |  |
| 5. | Do your neighbors, colleagues or others in your community have less respect for you because of this problem? |  |  |  |  |  |
| 6. | Do you think that contact with you might have any bad effects on others around you even after you have been treated? |  |  |  |  |  |
| 7. | Do you feel others have avoided you because of this problem? |  |  |  |  |  |
| 8. | Would some people refuse to visit your home because of this condition even after you have been treated? |  |  |  |  |  |
| 9. | If they knew about it would your neighbors, colleagues or others in your community think less of your family because of this problem? |  |  |  |  |  |
| 10. | Do you feel that your problem might cause social problems for your children in the community? |  |  |  |  |  |
| 11A | Do you feel that this disease has caused problems in getting married?  *(Unmarried only)* |  |  |  |  |  |
| 11B | Do you feel that this disease has caused problems in your marriage? *(Married only)* |  |  |  |  |  |
| 12. | Do you feel that this disease makes it difficult for someone else in your family to marry? |  |  |  |  |  |
| 13. | Have you been asked to stay away from work or social groups? |  |  |  |  |  |
| 14. | Have you decided on your own to stay away from work or social group? |  |  |  |  |  |
| 15. | Because of leprosy people think you also have other health problems? |  |  |  |  |  |
