# Supplementary material for: Factors Affecting Perceived Stigma in Leprosy Affected Persons in Western Nepal
Source: PLoS Negl Trop Dis. 2014 Jun 5;8(6):e2940. doi: 10.1371/journal.pntd.0002940 (PMC4046961; doi:10.1371/journal.pntd.0002940)
Supplement: Appendix S2 — Questionnaire for focus group discussion. (DOCX) [file pntd.0002940.s002.docx]

**Appendix S2: Questionnaire for focus group discussion**

Outlines/Questions:

1. Why do most of the leprosy affected people do not prefer to confess their disease to others?

2. Why do people affected with leprosy think they are less than others?

3. What do you think the reasons for being less respected when you have leprosy?

4. Do you think leprosy can have negative impact on marriage? if so, what might be the reasons ?

5. Have you got any bitter experiences in society which can be shared?
