# Supplementary material for: Factors Affecting Perceived Stigma in Leprosy Affected Persons in Western Nepal
Source: PLoS Negl Trop Dis. 2014 Jun 5;8(6):e2940. doi: 10.1371/journal.pntd.0002940 (PMC4046961; doi:10.1371/journal.pntd.0002940)
Supplement: Checklist S1 — STROBE checklist for cross-sectional studies. (DOC) [file pntd.0002940.s003.doc]

CHECKLIST S1: STROBE Checklist for cross-sectional studies

|  | Item No | Recommendation |
| --- | --- | --- |
| **Title and abstract** | 1 | (*a*) Indicate the study’s design with a commonly used term in the title or the abstract  ***Background:*** *The main purpose of this study was to determine the level of perceived stigma and the risk factors contributing to it among leprosy affected person attending the Green Pastures Hospital, Pokhara municipality of western Nepal.* ***Methods:*** *cross-sectional study was conducted among 135 people affected by leprosy at Green Pastures Hospital and Rehabilitation Centre* |
|  |  | (*b*) Provide in the abstract an informative and balanced summary of what was done and what was found  ***Methods:*** *A* *cross-sectional study was conducted among 135 people affected by leprosy at Green Pastures Hospital and Rehabilitation Centre. Persons above the age of 18 were interviewed using a set of questionnaire form and Explanatory Model Interview Catalogue (EMIC). In addition, two sets of focused group discussions each containing 10 participants from the ward was conducted with the objectives of answering the frequently affected EMIC items.*  ***Results:*** *Among 135 leprosy affected persons, the median score of perceived stigma was 10 while it ranged from 0-34. Higher perceived stigma score was found in illiterate persons (p=0.008), participants whose incomes were self-described as inadequate (p=0.014) and who had changed their occupation due to leprosy (p=0.018). Patients who lacked information on leprosy (p=0.025), knowledge about the causes (p=0.02) and transmission of leprosy (p=0.046) and those who had perception that leprosy is a severe disease (p<0.001) and is difficult to treat (p<0.001) had higher perceived stigma score. Participants with disfigurement or deformities (p=0.014), ulcers (p=0.022) and odorous ulcers (p=0.043) had higher perceived stigma score.* |
| Introduction | | |
| Background/rationale | 2 | Explain the scientific background and rationale for the investigation being reported  ***Introduction:*** *Leprosy is a chronic granulomatous disease caused by Mycobacterium leprae. Besides clinical sequel followed usually after infection, the consequences of stigma associated with leprosy outweigh the burden of physical afflictions [1]. Three kinds of stigma associated with leprosy affected persons have been described. Experienced or enacted stigma refers to the real discrimination or acts experienced by leprosy affected persons while perceived stigma refers to the development of fear within an affected person where the fear may arise out of potential discrimination from family members, friends or society. As a consequence of both enacted and perceived stigma, a person over a long period of time may believe what others think and say about him, resulting to the loss of self-esteem and dignity which is referred to be a self-stigma or internalized stigma [2].*  *Stigma affects the psychosocial well-being of the affected person. A person may feel fear or shame which can lead to anxiety and depression. The resultant anxiety and depression may lead to decreased social participation and social exclusion [3].*  *Anticipation of stigma may cause affected person to conceal their condition [4]. The burden of keeping this secret, of being ever watchful and careful takes an emotional toll and adversely affects health seeking behavior [3]. Concealing the disease, avoiding the questions regarding the disease and at times even telling lie for the fear of disclosure was found to be a major concern for leprosy affected persons attending Green Pastures Hospital, Nepal [5].*  *Stigma has been found to be associated with misconceptions about the disease, visible deformities and the development of ulcers [4]. Disability is a broad term covering any impairment, activity limitation or participation restriction affecting a person. According to WHO, grade 0 means no disability is found. Grade I means that loss of sensation has been noted in the hand or foot while grade II means the visible damage or disability is noted [6]. Visible deformities and disabilities have been found to be the prominent contributor of stigma development in leprosy affected persons [7] while it also triggers the development of negative attitudes towards leprosy among unaffected people [8].*  *In a systematic review of risk factors contributing to stigma, the basis of stigma development was found to be the visibility of the disfigurements and disability augmented by the stereotypes of the society, knowledge and the status of the person in terms of economy, education and ability to participate in society [9].*  *In Nepal, leprosy is still a stigmatizing disease. Misconceptions about the disease have contributed to the development of negative attitudes to leprosy affected persons. In a study conducted in eastern Nepal, fear of infection and god’s curse were found to be the most prevalent causes of negative behavior towards leprosy affected persons [8]. In the other study [10] conducted in eastern part of Nepal, the causes of stigma perception in leprosy affected persons were consistent with the causes of negative attitudes in unaffected community members [8]. The beliefs and perceptions about leprosy were found to be the prominent causes of stigma [10]. Fear of infection, was the most important cause of stigma different countries including China [11] and India [12]. In India, in addition to the fear of infection, false beliefs about leprosy, ignorance about the disease and lower socio-economic status were associated with stigma in leprosy [12]. Therefore, we hypothesized that there is association between the levels of perceived stigma in leprosy affected persons and the factors characterizing them (demographic characteristics, knowledge about leprosy, natural history of disease, clinical presentation, disability grades and reaction) While few studies are done in eastern part of Nepal, most of them are focused on the impact of the stigma, participation restriction and income generation. There has been no research so far in leprosy stigma in a view to explore the factors associated with it. The specific objective of this study was to determine the prevalence of perceived stigma and its association with factors such as socio-demographic, knowledge about leprosy and clinical presentation characterizing leprosy affected persons attending Green Pastures Hospital and Rehabilitation Centre.*  *Green Pastures Hospital and Rehabilitation Centre, the only known leprosy referral center in western region of Nepal provides the services for leprosy patients with disability management, treatment and vocational training. Therefore, exploring the risk factors of stigma in leprosy affected persons attending GPH&RC can help to understand the leprosy stigma and therefore can direct the stigma reduction strategies and intervention programs.* |
| Objectives | 3 | State specific objectives, including any pre-specified hypotheses  ***Introduction (6^th^ paragraph/2^nd^ last paragraph)*** *Therefore, we hypothesized that there is association between the levels of perceived stigma in leprosy affected persons and the factors characterizing them (demographic characteristics, knowledge about leprosy, natural history of disease, clinical presentation, disability grades and reaction) While few studies are done in eastern part of Nepal, most of them are focused on the impact of the stigma, participation restriction and income generation. There has been no research so far in leprosy stigma in a view to explore the factors associated with it. The specific objective of this study was to determine the prevalence of perceived stigma and its association with factors such as socio-demographic, knowledge about leprosy and clinical presentation characterizing leprosy affected persons attending Green Pastures Hospital and Rehabilitation Centre.* |
| Methods | | |
| Study design | 4 | Present key elements of study design early in the paper  ***Materials and Methods:*** *The study was cross-sectional in design. The study population comprised leprosy affected people attending Green Pastures Hospital and Rehabilitation Centre, specific for the treatment of leprosy and rehabilitation in western region of Nepal.* |
| Setting | 5 | Describe the setting, locations, and relevant dates, including periods of recruitment, exposure, follow-up, and data collection  ***Materials and Methods:*** *The study sample comprised people affected by leprosy who were undergoing treatment for leprosy reactions, ulcers, disability-rehabilitation in addition to self-care training at Green Pastures Hospital and Rehabilitation Centre. All participants were included in the study who visited the hospital between the periods of February 2013 to March 2013. Considering the limited number of people affected by leprosy visiting the hospital, pilot testing of the questionnaire was not conducted.*  *Focus group discussions were conducted with 20 people affected with leprosy who were admitted in ward. Semi-structured questionnaire were designed to explore the deeper reasons for the most affected EMIC domains which were reasons of concealment, lower self-esteem, less respect from others, impacts on marriage and their experiences with leprosy. Out of 42 people who were admitted in ward during that period, only 20 of them agreed to participate in 2 sets of focus group discussion each containing 10 participants. An oral consent was taken with each participant before they took part in discussion.* |
| Participants | 6 | (*a*) Give the eligibility criteria, and the sources and methods of selection of participants  ***Ethics Statement***  *Ethical permission for this research was obtained from Nepal Health Research Council and International Nepal Fellowship Research Committee. People were eligible if they were affected by leprosy, age above 18 years and willing to participate. Interviews were only conducted after the written consent was received and was conducted by principal investigator. Interviews were conducted with all leprosy affected people attending GPH&RC from February 2013 to March 2013. Attempt was done to include equal number of participants from the ward and OPD, 5 from the ward and 3 from the OPD denied the written consent, however, there were no drop outs. The interviewer taking into the consideration the sensitivity of the subject established a friendly rapport before the interview and encouraged participants to express their views. The anonymity of the participants was secured by coding the participants’ name. No incentives were offered or paid for their time.* |
| Variables | 7 | Clearly define all outcomes, exposures, predictors, potential confounders, and effect modifiers. Give diagnostic criteria, if applicable  *Total 135 leprosy affected persons were interviewed using a questionnaire containing socio-demographic characteristics (age, sex, ethnicity, marital status, location, type of family and leprosy affected persons in family/relatives/neighbors), socio-economic conditions (occupation, income, nature of work, job, education and religion), Knowledge about leprosy (information about leprosy, cause of leprosy, infectiousness, transmission, treatment, signs and symptoms about leprosy) and Clinical presentation of leprosy (ulcer, disfigurement, deformity and disability status). Clinical conditions such as ulcer, disfigurement, deformity and disability grades were obtained from the hospital treatment card which individual participants carried with them. Knowledge about leprosy was assessed using questions with answers as yes or no and corresponding sub-questions on further knowledge regarding the particular items.*  *In addition, the EMIC scale questionnaire was asked to each participant. The EMIC scale has been developed to elicit illness-related perceptions, beliefs and the practices [13]. The EMIC questionnaire has 15 items related to perception of stigma in leprosy and has been validated and shown to be reliable in a study in India [14]. EMIC scale has been available in different languages including Nepali language and is the recommended instrument in terms of measuring leprosy related stigma. Higher the score obtained by EMIC scale higher is the level of perceived stigma. It has been classified as the instrument to measure the perceived stigma in leprosy by The International Federation of Anti-Leprosy Association (ILEP) and the stigma research workshop held in Amsterdam in 2010 [15, 16].* |
| Data sources/ measurement | 8* | For each variable of interest, give sources of data and details of methods of assessment (measurement). Describe comparability of assessment methods if there is more than one group  *Total 135 leprosy affected persons were interviewed using a questionnaire containing socio-demographic characteristics (age, sex, ethnicity, marital status, location, type of family and leprosy affected persons in family/relatives/neighbors), socio-economic conditions (occupation, income, nature of work, job, education and religion), Knowledge about leprosy (information about leprosy, cause of leprosy, infectiousness, transmission, treatment, signs and symptoms about leprosy) and Clinical presentation of leprosy (ulcer, disfigurement, deformity and disability status). Clinical conditions such as ulcer, disfigurement, deformity and disability grades were obtained from the hospital treatment card which individual participants carried with them. Knowledge about leprosy was assessed using questions with answers as yes or no and corresponding sub-questions on further knowledge regarding the particular items.*  *In addition, the EMIC scale questionnaire was asked to each participant. The EMIC scale has been developed to elicit illness-related perceptions, beliefs and the practices [13]. The EMIC questionnaire has 15 items related to perception of stigma in leprosy and has been validated and shown to be reliable in a study in India [14]. EMIC scale has been available in different languages including Nepali language and is the recommended instrument in terms of measuring leprosy related stigma. Higher the score obtained by EMIC scale higher is the level of perceived stigma.* |
| Bias | 9 | Describe any efforts to address potential sources of bias  *Clinical conditions such as ulcer, disfigurement, deformity and disability grades were obtained from the hospital treatment card which individual participants carried with them. Thus the potential recall bias was avoided.* |
| Study size | 10 | Explain how the study size was arrived at  *All participants were included in the study who visited the hospital between the periods of February 2013 to March 2013.* |
| Quantitative variables | 11 | Explain how quantitative variables were handled in the analyses. If applicable, describe which groupings were chosen and why  *Descriptive statistics were used to describe the socio-economic, knowledge level and clinical presentation of the participants. Difference in total perceived stigma score using EMIC between different categorical variables were analyzed using Mann Whitney U test and Kruskal Wallis H test since these scores were not normally distributed.* |
| Statistical methods | 12 | (*a*) Describe all statistical methods, including those used to control for confounding  *Descriptive statistics were used to describe the socio-economic, knowledge level and clinical presentation of the participants. Difference in total perceived stigma score using EMIC between different categorical variables were analyzed using Mann Whitney U test and Kruskal Wallis H test since these scores were not normally distributed.* |
|  |  | (*b*) Describe any methods used to examine subgroups and interactions |
|  |  | (*c*) Explain how missing data were addressed  *There were no missing data.* |
|  |  | (*d*) If applicable, describe analytical methods taking account of sampling strategy |
|  |  | (*e*) Describe any sensitivity analyses |
| Results | | |
| Participants | 13* | (a) Report numbers of individuals at each stage of study—eg numbers potentially eligible, examined for eligibility, confirmed eligible, included in the study, completing follow-up, and analysed  *All participants who met the eligibility criteria were recruited into the study after taking written consent.* *Total 135 participants were asked with the questionnaire form. Similarly, Focus group discussions were conducted with 20 people affected with leprosy who were admitted in ward. Semi-structured questionnaire were designed to explore the deeper reasons for the most affected EMIC domains which were reasons of concealment, lower self-esteem, less respect from others, impacts on marriage and their experiences with leprosy. Out of 42 people who were admitted in ward during that period, only 20 of them agreed to participate in 2 sets of focus group discussion each containing 10 participants. An oral consent was taken with each participant before they took part in discussion.*  *Both sets of focus group discussion were recorded in mobile phone recorder later typed into computer. Different themes according to the questionnaire were segregated and frequencies of themes were based as evidence for the formulation of conclusion* |
|  |  | (b) Give reasons for non-participation at each stage |
|  |  | (c) Consider use of a flow diagram |
| Descriptive data | 14* | (a) Give characteristics of study participants (eg demographic, clinical, social) and information on exposures and potential confounders  *It has been detailed in* ***Result*** *section*  *Of the 135 leprosy affected participants, 58.5% of them were those who attended OPD at the hospital. Total median score of EMIC scale was higher among those leprosy patients who were in the ward compared to those who attended OPD (p=0.006).* |
|  |  | (b) Indicate number of participants with missing data for each variable of interest |
| Outcome data | 15* | Report numbers of outcome events or summary measures  *Outcome has been presented in each result section with following headings: Socio-demographic characteristics, Knowledge about leprosy and perceived stigma score, History of disease, clinical presentation and perceived stigma score, WHO disability grading and perceived stigma scores, Focus Group Discussion* |
| Main results | 16 | (*a*) Give unadjusted estimates and, if applicable, confounder-adjusted estimates and their precision (eg, 95% confidence interval). Make clear which confounders were adjusted for and why they were included |
|  |  | (*b*) Report category boundaries when continuous variables were categorized |
|  |  | (*c*) If relevant, consider translating estimates of relative risk into absolute risk for a meaningful time period |
| Other analyses | 17 | Report other analyses done—eg analyses of subgroups and interactions, and sensitivity analyses  ***Result***  *On post hoc analysis, the illiterate and those who attended more than 5years of education had significant difference in median score (p=0.03). Similarly, when EMIC scores among subjects with less than 5 years education were compared with those with more than 5 years there was a significant difference (p=0.016) while EMIC scores of the illiterate and those who attended <5 years of education were not significantly different (p=0.673).*  *However, on post hoc analysis two different income groups (the highest and lowest income group) showed significant difference (p=0.011)* |
| Discussion | | |
| Key results | 18 | Summarise key results with reference to study objectives  *Key results have been meticulously discussed with references.* |
| Limitations | 19 | Discuss limitations of the study, taking into account sources of potential bias or imprecision. Discuss both direction and magnitude of any potential bias  ***Limitations***  *This study was conducted in western region of Nepal, where only those people who visited hospital for treatment, rehabilitation and wound care were recruited while many other people affected by leprosy who did not have any symptoms were not included in the study which limits our finding to generalize over all leprosy affected persons. Only perceived stigma was assessed in this study while two other types of stigma were not assessed therefore, stigma in this study cannot be the whole picture of stigma. While clinical presentations of the participants were obtained from the hospital treatment card, many other questions might have encountered recall biases. The full evaluation of the data using multiple regressions was not done in this study which could have strengthened our findings.* |
| Interpretation | 20 | Give a cautious overall interpretation of results considering objectives, limitations, multiplicity of analyses, results from similar studies, and other relevant evidence  ***Conclusion***  *This study concludes that lower education level, perceived economic inadequacy, obligation to change the occupation due to leprosy, lack of knowledge and the wrong perceptions about leprosy were the significant factors contributing to higher levels of perceived stigma in leprosy affected persons. In addition to these socio-demographic factors, the presence of visible deformities, ulcers and disabilities also contributed to higher perceived stigma in leprosy affected persons. The major aspects of EMC stigma scale affected were the attitude to conceal the disease, and lowered self-esteem. The major causes for these have been explained by focus group discussion as the perceived fear of discrimination, rejection and the society’s fear of transmission.*  *The factors contributing to the development of stigma in leprosy affected persons from this study can direct the need of intervention programs focusing on health education. Health education which might correct the wrong perceptions and might increase understanding of leprosy and the people affected can have a significant impact in both leprosy affected persons and leprosy unaffected persons. In addition to the education and health awareness programs, empowerment of the leprosy affected persons by technical education, vocational training and social participation might be helpful to increase self-esteem and reduce perceived stigma.*  *Ulcers and visible deformities have been found as contributing factors for the higher level of perceived stigma. Early case detection through training of health professionals and health education to the general public might prevent the delays in presentation, ulcers, and deformities which ultimately can reduce the stigma.* |
| Generalisability | 21 | Discuss the generalisability (external validity) of the study results  *This study was conducted in western region of Nepal, where only those people who visited hospital for treatment, rehabilitation and wound care were recruited while many other people affected by leprosy who did not have any symptoms were not included in the study which limits our finding to generalize over all leprosy affected persons. Only perceived stigma was assessed in this study while two other types of stigma were not assessed therefore, stigma in this study cannot be the whole picture of stigma.* |
| Other information | | |
| Funding | 22 | Give the source of funding and the role of the funders for the present study and, if applicable, for the original study on which the present article is based  *No funding was available for this study.* |

*Give information separately for exposed and unexposed groups.

**Note:** An Explanation and Elaboration article discusses each checklist item and gives methodological background and published examples of transparent reporting. The STROBE checklist is best used in conjunction with this article (freely available on the Web sites of PLoS Medicine at http://www.plosmedicine.org/, Annals of Internal Medicine at http://www.annals.org/, and Epidemiology at http://www.epidem.com/). Information on the STROBE Initiative is available at www.strobe-statement.org.
